# Efficacy of Platelet-Rich Plasma, Mesenchymal Stromal Cells, and Hyaluronic Acid in Preventing Adhesions After Zone II Flexor Tendon Repair: A Narrative Review

**DOI:** 10.7759/cureus.104663

**Published:** 2026-03-04

**Authors:** Garrett J Rutt, Chloe E Esch, Marcia Ballantyne

**Affiliations:** 1 Medicine, Lake Erie College of Osteopathic Medicine, Bradenton, USA; 2 Physical Medicine and Rehabilitation, Lake Erie College of Osteopathic Medicine, Bradenton, USA; 3 Pathology, Lake Erie College of Osteopathic Medicine, Bradenton, USA

**Keywords:** mesenchymal stromal cells, plasma-rich platelets, post-operative adhesions, zone ii flexor tendon lacerations, zone ii flexor tendon repair, hyaluronic acid

## Abstract

Zone II flexor tendon injuries remain among the most challenging conditions in hand surgery due to the region’s complex anatomy and high rates of postoperative complications, particularly adhesion formation and the resulting loss of range of motion. Although platelet-rich plasma (PRP), mesenchymal stromal cell (MSC) therapies, and hyaluronic acid (HA) have demonstrated therapeutic potential in other musculoskeletal conditions, their effectiveness in preventing postoperative adhesions following zone II flexor tendon repair, specifically, remains unclear. A comprehensive review of the literature was conducted using PubMed and Google Scholar to identify randomized controlled trials, prospective and retrospective cohort studies, and original research evaluating the use of PRP, MSCs, or HA in zone II flexor tendon repair. Only full-length English-language studies specifically addressing zone II injuries were included. Studies were excluded if they did not focus explicitly on zone II flexor tendon injuries, lacked clearly reported outcomes, or failed to assess postoperative adhesion formation and functional recovery. Search terms included but were not limited to “Zone II flexor tendon,” “PRP Zone II flexor tendon repair,” “stem cell Zone II flexor tendon repair,” “hyaluronic acid Zone II flexor tendon repair." Data from both human and animal studies were collected to compare postoperative adhesion formation and functional outcomes. Studies across all compounds showed inconsistent results in animal and human trials. PRP for zone II flexor tendon repair showed limited and inconsistent benefits, with most preclinical and clinical trials reporting no significant improvement in adhesion prevention or range of motion. Higher leukocyte concentrations in PRP were associated with increased inflammatory activity, potentially promoting scar formation. In contrast, mesenchymal stromal cell therapies, particularly adipose- and synovium-derived MSCs, demonstrated promising preclinical effects by modulating inflammation, reducing scar-related gene expression, limiting apoptosis, and enhancing tendon gliding though statistical significance was not always achieved. HA showed the most promising results with a majority of animal and human studies consistently reducing adhesion formation and improving tendon excursion and functional outcomes in the long-term. Across all the examined studies, no agent- PRP, MSCs, or HA- consistently prevented postoperative adhesions or reliably improved range of motion following zone II flexor tendon repair, specifically. The complex anatomy of zone II, inconsistent functional outcomes, limited mechanistic understanding, and wide methodological variation among studies, continue to limit the ability to draw definitive conclusions. Cost and accessibility further complicate the clinical adoption of these adjuncts. Current evidence does not support the routine use of PRP, MSCs, or HA for preventing postoperative adhesions after zone II flexor tendon repair. Nevertheless, while research on this topic remains limited, a few existing studies have shown promising trends that warrant further investigation. Future research should incorporate age-specific analyses, standardized agent formulations, clearer mechanistic evaluation, and optimized delivery methods to better determine whether these adjuncts can consistently and reliably improve outcomes in this anatomically challenging region.

## Introduction and background

Anatomy

Zone II of the volar hand, often referred to as “no man’s land,” is notoriously challenging to manage due to its complex anatomy and the high risk of postoperative complications. This zone has a fibro-osseous digital canal where both tendons interweave. Additionally, the multiple pulleys increase its complexity, as minimal swelling of the epitenon can impair free motion of the tendon. Any infection, fibrosis, cicatrix, overcrowding, etc., following surgical intervention can lead to dense adhesions, compromising postoperative outcomes; consequently, the margin of error in this zone is very small [1]. Tendon injuries in zone II frequently result in adhesions between the flexor tendons and surrounding soft tissues, leading to suboptimal rehabilitation outcomes. As mentioned earlier, zone II encompasses both the flexor digitorum superficialis (FDS) and flexor digitorum profundus (FDP) tendons as they course through the fibro-osseous sheath from the A1 pulley to the FDS insertion. For treatment planning, it can be subdivided into three regions: zone IIa, where the FDS lies inferior to the FDP; zone IIb, where the FDS is positioned laterally; and zone IIc, where the FDS lies superior to the FDP. Adhesion formation is greatest in areas with minimal differential tendon gliding within the sheath, and it is therefore believed that the most substantial adhesions tend to occur beneath the A2 and A3 pulleys [2]. Due to this region's anatomic complexity, Tobler-Ammann et al. found that patients with zone II injuries experienced significantly poorer range of motion (ROM) compared to those with zone III injuries, along with higher rates of flexion contractures, tendon ruptures, and the need for local anesthesia. The most frequently reported postoperative concerns included diminished mobility, sensory deficits, and reduced dexterity, issues that were particularly prevalent following zone II repairs [3]. 

Surgical approaches

Given the high complication rate associated with zone II flexor tendon repairs, numerous studies have explored optimal surgical strategies to improve functional outcomes and minimize postoperative adhesions. However, no single technique has consistently demonstrated superiority in reducing adhesion formation or preventing motion limitations. Kotwal et al. discussed the standard midlateral incision or Bruner's zigzag incision as it prevents scar formation directly over the tendon and is less likely to break down during physiotherapy; however, it requires surgical dissection directly over the neurovascular bundle and is therefore a surgically demanding procedure. Once adequate exposure is achieved, the authors outlined three principal treatment options: (1) repair of the FDP tendon alone with debridement of the FDS stump; (2) repair of both tendons; or (3) repair of the FDP with reconstruction of a single slip of the FDS. Although repairing both tendons is ideal for restoring function, it is more demanding in zone II due to the anatomic complexity mentioned earlier [1]. A survey of 290 orthopedic surgeons and hand specialists further highlighted the wide range of surgical approaches, including the use of Modified Kessler, Adelaide, and Double Modified Kessler techniques; decisions regarding repair of the FDP and/or FDS; incorporation of an epitendinous suture; and selection of suture caliber (3-0, 4-0, or 5-0) [4]. Additional approaches described in the literature include but are not limited to externalized detensioning sutures, four- and six-strand repair constructs, and the Strickland repair augmented with a running epitendinous stitch [5-7]. 

Rehabilitation protocols 

In addition to the ongoing debate regarding the optimal surgical technique for minimizing postoperative adhesions and motion loss, there is also considerable uncertainty on the most effective rehabilitation strategy for zone II flexor tendon repairs. While the literature encompasses a wide range of rehabilitation protocols, the common goals of all rehabilitation regimens after tendon repair are to promote intrinsic tendon healing and minimize extrinsic scarring to optimize tendon gliding and functional ROM while also avoiding complications of re-rupture, adhesion formation, flexion contracture, pulley failure/bowstringing, and triggering [1]. Some proposed rehabilitation regimens include early active flexion combined with controlled passive motion, early controlled passive motion alone (such as the modified Kleinert protocol), three weeks of immobilization, place-and-hold exercises within an orthosis, and controlled passive motion using rubber-band traction. Unfortunately, evidence comparing these approaches shows very low certainty of a superior approach, with minimal differences in self-reported function or the incidence of postoperative adhesions [8]. A prospective study of 145 digits with complete FDP lacerations in zone II evaluated recovery of active interphalangeal (IP) joint motion over a one-year follow-up period. The authors found no statistically significant differences in final outcomes among the three rehabilitation approaches: the Kleinert regimen, the four-finger method, and controlled mobilization. Overall, the study reported that patients recovered an average of 37% of their final distal interphalangeal (DIP) joint motion and 9% of their final proximal interphalangeal (PIP) joint motion between three months and one year postoperatively [9].

Biologic therapies in zone II flexor tendon repair

Given the absence of conclusive evidence supporting a single surgical technique or rehabilitation regimen to reliably prevent postoperative adhesions and restore optimal ROM following zone II flexor tendon repair, further investigation is clearly warranted. Therapies such as platelet-rich plasma (PRP), mesenchymal stromal cell (MSC) treatments, and hyaluronic acid (HA) have been applied in various conditions including lateral epicondylitis, knee osteoarthritis, patellar tendinopathy, plantar fasciitis, carpal tunnel syndrome, and rotator cuff pathology [10-12]. Their success in these musculoskeletal applications has prompted growing interest in evaluating their potential to enhance healing, improve ROM, and reduce adhesion formation following zone II flexor tendon repair. Although multiple mechanisms have been proposed for the effects of PRP, MSC therapy, and HA, a common theme across all three appears to be their capacity to modulate the immune response to varying degrees. 

Platelet-Rich Plasma (PRP)

PRP has shown mixed results in tendon repair and healing, although its proposed mechanism centers on delivering a concentrated platelet suspension to injury sites, promoting the release of growth factors and adhesion proteins, initiating the hemostatic cascade, stimulating connective tissue synthesis, and enhancing revascularization [13]. One study demonstrated that PRP can promote cell migration, proliferation, and tenogenic differentiation through upregulation of scleraxis (SCX), Collagen Type I Alpha 1 chain (COL1A1), and vascular endothelial growth factor (VEGF), suggesting a potential benefit during early tendon repair [14]. A review of both in vitro and in vivo studies highlighted multiple mechanisms through which PRP may support tendon healing, while also emphasizing the variability and inconclusiveness of results. Among studies reporting positive effects, mechanisms included increased fibroblast and tenocyte proliferation, enhanced collagen synthesis and organization, improved cell viability and migration, and reduced apoptosis, processes mediated by growth and angiogenic factors such as VEGF, epidermal growth factor (EGF), platelet-derived growth factor (PDGF), collagen type 1 (COL1), and cartilage oligomeric matrix protein (COMP) [15]. Despite these findings, the overall evidence remains inconclusive, and no standardized PRP formulation exists to reliably achieve these therapeutic effects [13]. It is believed that the composition of white blood cells (WBCs) within a PRP formulation is one of the most crucial factors as a controlled inflammatory response is essential for proper healing; however, excessive inflammation can exacerbate scar formation and adhesion development, ultimately compromising postoperative ROM [16].

Mesenchymal Stromal Cells (MSCs)

MSCs are being investigated for the treatment of tendon and soft tissue injuries, though a universally accepted mechanism of action has yet to be established. One review reported that MSCs primarily exert their effects through the secretion of paracrine factors, including extracellular vesicles and cytokines, the transfer of mitochondria to neighboring cells, and modulation of the immune response, all of which protect injured tissue and promote endogenous regeneration. Mechanistically, these actions are associated with upregulation of signal transducer and activator of transcription 3 (STAT3), VEGF, and extracellular signal-regulated kinase/protein kinase B (ERK/Akt) signaling, along with downregulation of pro-inflammatory cytokines such as tumor necrosis factor (TNF)-α, IL-1, IL-6, and apoptotic pathways [17]. Similar to PRP, a mechanistic understanding and clinical efficacy remain limited, leaving their effect on postoperative ROM and adhesions after zone II flexor tendon repair unclear. An additional challenge in MSC-based therapy is optimizing cell expansion in vitro to avoid aneuploidy, which could increase the risk of cancer development or progression in vivo [17]. A second group of researchers discussed two potential mechanistic theories for MSC effectiveness: differentiation theory and paracrine theory. The differentiation theory states that chondrocytes replace the damaged cells by dividing and proliferating within the area or by secreting extracellular matrix (ECM) components to aid in tissue regeneration. The mechanism is composed of mesenchymal condensation, proliferation and differentiation of chondrocytes. The paracrine theory, similar to the prior study, proposes that MSCs promote repair not by directly becoming new tissue, but by releasing bioactive molecules and extracellular vesicles that shape the local environment for healing. These secreted factors recruit and guide endogenous MSCs to differentiate into chondrocytes, support chondrocyte maintenance, and modulate immunity by shifting macrophages from a pro-inflammatory (M1) to an anti-inflammatory (M2) state, thereby reducing IL-1 and IL-6 levels and limiting tissue damage [18].

Hyaluronic Acid (HA)

HA has also demonstrated therapeutic potential in the treatment of tendon and soft-tissue injuries, though its precise mechanism of action remains unknown [19,20]. Current evidence supports three primary pathways by which HA may reduce adhesion formation. First, HA directly facilitates tendon healing by binding to HA-binding proteins on fibroblasts, promoting their migration to the injury site and supporting nutrient exchange essential for cell proliferation, collagen synthesis, and matrix secretion [21,22]. Second, high-molecular weight HA decreases peritendinous adhesion formation by inhibiting granulocyte phagocytosis and transmigration, which reduces inflammation at the tendon and paratenon interface [23,24]. A recent hypothesis proposes that synovium-derived mesenchymal stem cells (SMSCs) may help prevent postoperative adhesions following zone II flexor tendon repair. SMSCs are multipotent and exhibit a higher proliferative capacity than other MSC populations. Due to this, they are believed to also demonstrate increased expression of the has2 gene, which is associated with the production of high-molecular-weight HA. Because HA is known to reduce friction and inhibit adhesion formation, the authors suggest that SMSCs may serve as an effective therapeutic candidate for minimizing peritendinous adhesions after zone II digital flexor tendon surgery [24]. Third, HA functions as a biological barrier, physically separating the injured tendon from surrounding tissues and limiting unwanted contact that can lead to adhesion development [25].

Current limitations

PRP, MSCs, and HA therapies show significant potential for treating various tendinopathies and supporting tendon repair, but their mechanisms, clinical evidence, and efficacy remain limited. Notably, studies demonstrating positive effects have not specifically addressed zone II flexor tendon lacerations and existing literature investigating surgical techniques, suture materials, and rehabilitation programs have yielded inconclusive results. Given the anatomic and surgical complexity and high rate of postoperative complications in Zone II repairs, further research is needed. With the growing use of PRP, MSCs, and HA in other tendon pathologies, their application to Zone II flexor tendon repair warrants further investigation. This review summarizes the available animal and human literature evaluating PRP, MSCs, and HA therapies for improving postoperative ROM and reducing adhesions following zone II flexor tendon repairs.

## Review

Methods

This narrative review examines the potential therapeutic roles of PRP, MSCs, and HA in reducing postoperative adhesions and improving the ROM following zone II flexor tendon repair. To our knowledge, this is the first focused, up-to-date synthesis addressing biologic adjuncts specifically in the context of Zone II flexor tendon injury and repair. Relevant literature was identified through nonsystematic searches of PubMed and Google Scholar. Search terms included “Zone II flexor tendon,” “PRP Zone II flexor tendon repair,” “stem cell Zone II flexor tendon repair,” “hyaluronic acid Zone II flexor tendon repair,” “PRP,” “stem cell therapy,” and “hyaluronic acid therapy.” Searches were conducted intermittently, with the most recent search completed in November 2025.

To maintain relevance to the topic, we considered full-length clinical trials, randomized controlled trials, original research studies, and animal studies published in English between 1992 and 2022. Studies were excluded if they did not clearly involve Zone II or intrasynovial flexor tendon injuries, if results were unclear or unpublished, if the publication had been retracted, or if the article was not available in English. Approximately 158 articles were initially identified. Of these, 12 studies met the inclusion criteria and did not meet any exclusion criteria, and were therefore selected for review. An additional study was included based on its clinical relevance, bringing the total to 13 studies. Key methodological characteristics and outcome measures related to postoperative ROM and adhesion formation were extracted from the included studies and systematically summarized. Comparative tables were developed to highlight findings for PRP, MSCs, and HA, alongside their respective control groups.

The screening and synthesis process was conducted collaboratively. All authors reviewed the identified literature, and data extraction was independently verified by all authors. In cases of unclear results or disagreements, the research team engaged in discussion until a consensus was reached.

Results

PRP

A randomized controlled trial [26] was performed to evaluate the intraoperative application of PRP on 37 New Zealand White rabbits, weighing 3.0-3.5 kg, with zone II flexor tendon lacerations (Table 1).

**Table 1:** Characteristics of the in vivo animal studies using PRP The table summarizes the outcome measures and key findings from animal studies evaluating the efficacy of PRP in preventing postoperative adhesions following zone II flexor tendon repair. Results were considered statistically significant if the p-value <0.05. PRP: platelet-rich plasma; ROM: range of motion; PIP: proximal interphalangeal joint; MCP: metacarpophalangeal joint.

Study	Species/ Sample Size	Intervention	Outcomes Measured	Key Findings
Kollitz et al. (2014) [26]	New Zealand White rabbits, n=37	0.5 mL autologous PRP applied to the repair site, intraoperatively	Tendon strength, ROM (PIP, MP, total)	No statistically significant differences in excursion or ROM.
Sato et al. (2012) [27]	Japanese White rabbits, n=73	PRP (0.5 μL), fibrin (1 µL), PRP+fibrin (0.5 μL each) applied before closure of epitendinous suture	Edema score, Adhesion score	No statistically significant differences in edema or adhesion formation.

All rabbits in each treatment group underwent the same pre- and postoperative procedures; the only difference was 0.5 mL of autologous PRP applied to the repair site in the treatment group. The rabbits were then sacrificed two, four, or eight weeks postoperatively and evaluated for tendon strength and ROM, an indirect measurement of postoperative adhesions. The study reported that there was moderate reliability for PIP ROM, with a correlation coefficient of 0.66, and no statistically significant difference between time points or treatment groups for excursion, metacarpophalangeal (MCP) ROM, PIP ROM, or total ROM. Total ROM was noted to trend higher at two and eight weeks in the PRP group, but did not reach significance compared to the control group (p=0.29 and p=0.58, respectively) [26].

A second randomized controlled trial was conducted to evaluate the effects of different PRP formulations on 156 toe flexor tendons of 73 Japanese White rabbits (2.5-3.5 kg) with zone II flexor tendon lacerations (Table 1). Each rabbit received the same anesthetic and postoperative care. The rabbits were assigned to one of four treatment groups and underwent flexor digitorum fibularis tendon transection, between the A1 and A2 pulley, and immediately repaired with the same intratendinous suture repair method. The groups included a control group (n=38) receiving no adjunct treatment; a PRP group (n=25) receiving 0.5 μL of PRP; a fibrin group (n=23) receiving 1.0 μL of fibrin; and a PRP-fibrin group (n=50) receiving 0.5 μL each of PRP and fibrin. Each experimental group received their assigned PRP formulation at the repair site prior to completing the epitendinous suture. Each of the rabbits was then euthanized and their tendons were analyzed for edema and adhesions. Edema was scored based on the system as zero (swelling absent), one (minimal swelling), two (obvious swelling), or three (prominent swelling with open skin incision). Adhesions were evaluated as follows: zero (no adhesions apparent), one (adhesions extending halfway along zone II or halfway around the tendon), two (adhesions extending beyond halfway along zone II or beyond halfway around the tendon), or three (adhesions entirely along zone II or completely around the tendon). The investigators found no statistically significant differences in edema or peritendinous adhesion formation among the four groups at two-, three-, or six-week follow-ups [27].

To investigate applicability of PRP in humans with zone II flexor tendon repair, a prospective interventional controlled study [28] involving 40 participants was conducted and compared postoperative ROM, a measure related to postoperative adhesions (Table 2).

**Table 2:** Characteristics of the in vivo human studies using PRP The table summarizes the outcome measures and key findings from human studies evaluating the efficacy of PRP in preventing postoperative adhesions following zone II flexor tendon repair. Results were considered statistically significant if the p-value <0.05. PRP: platelet-rich plasma; ROM: range of motion; TAROM: total active range of motion.

Study / Year	Sample size	Intervention	Outcomes measured	Key findings
Ayman et al. (2021) [28]	n=40 patients	PRP injection at repair site at 1 and 6 weeks s/p repair	ROM (Buck-Gramcko II criteria)	No statistically significant difference in ROM.
Elfeshawy et al. (2022) [29]	n=40 patients	PRP injection into/around repair site	TAROM, Ultrasound analysis for adhesion formation (peritendonous scar, Doppler intensity, spindle shaped tendon, tendon excursion)	No statistically significant difference in TAROM. Statistically significant greater tendon excursion and vascularity (suggests decreased adhesion formation).

All participants underwent tendon repair with the same surgical technique, followed by equivalent physiotherapy; however, the experimental group was supplemented with a PRP injection around the tendon at the site of repair after one and six weeks. Researchers evaluated the ROM of both groups using the Buck-Gramcko II criteria at six and 12 weeks postoperatively. At six weeks, the physiotherapy group supplemented with PRP demonstrated a mean ROM score of 11.5 versus 10 in the control group. At 12 weeks, mean ROM was 14 in the PRP-supplemented group compared with 12.5 in control. While the study indicated a trend toward improved ROM with PRP supplementation, the results were not statistically significant (P=0.46, P=0.207, respectively) [28].

A second prospective controlled clinical trial investigating the effect of PRP was performed involving 40 patients with complete laceration of flexor tendons in zones two through six (Table 2). There was no significant age difference between groups of participants included in the study, with mean ages of 29.7 in the control group and 30.8 in the PRP group. Both groups included patients with various flexor tendon injuries, with six zone II lacerations in the control group and eight in the PRP group. All participants included in the study underwent the same preoperative testing, postoperative care, and tendon repair using the traditional double-modified Kessler technique with an orientated continuous suture. The experimental group also received an autologous PRP injection in and around the surgical site. The study assessed eight postoperative tendon-related outcomes; however, the two most relevant to this review were dynamic imaging of passive tendon movement (categorized as good vs. bad) and evaluation of soft tissue changes via ultrasound, including the presence of adhesions between the tendon and surrounding structures (good vs. bad) [29]. 

Researchers reported no statistically significant differences in total active range of motion (TAROM), a measure related to postoperative adhesion formation, between the control and PRP treatment groups at one (50° vs. 55°), two (55° vs. 65°), or three (60° vs. 70°) weeks postoperatively. Following ultrasound analysis, adhesion formation was inferred indirectly through several indicators, such as thickened peritendinous scar tissue, restricted gliding surfaces, Doppler signal intensity, persistence of a spindle-shaped tendon, and tendon excursion measurements, all of which correlate with tendon mobility and adhesion presence. The study found that a persistent spindle-like tendon shape on ultrasound and increased power Doppler signal were associated with better clinical outcomes and greater tendon excursion at three months, suggesting dominant intrinsic healing and a lower likelihood of adhesion formation. Ultrasound assessments indicated that 12 patients in the PRP group had “good” postoperative outcomes compared to eight in the traditional repair group, though this difference was not statistically significant. All repaired tendons exhibited a spindle-like shape on gray-scale ultrasound at two weeks. By 12 weeks, only 50% retained this shape, and those that did showed significantly greater tendon excursion (2.86 ± 0.61 mm in the PRP group vs. 1.14 ± 0.27 mm in the traditional group). Regarding vascularity, 50% of tendons demonstrated increased Doppler signal at two weeks, with 40% maintaining elevated vascularity at 12 weeks. Persistently elevated vascularity correlated with greater tendon excursion and improved dynamic movement (3.13 ± 0.70 mm in PRP vs. 1.25 ± 0.6 mm in traditional). Taken together, these findings suggest that PRP may enhance tendon ROM and reduce adhesion formation [29]. 

Unfortunately, a major limitation of Elfeshawy et al. is that while the authors reported the number of patients with zone II flexor tendon laceration in the demographic data, they did not provide subgroup-specific outcomes for this cohort. Consequently, the study could not confidently draw conclusions about the effects of PRP on postoperative adhesions specifically in zone II flexor tendon repairs [29].

An important consideration when using PRP in a given setting, let alone its use in preventing postoperative adhesions following zone II flexor tendon repair, is the optimal WBC composition. McCarrel et al. investigated the ideal WBC-to-platelet ratio in PRP formulations for tendon healing. Using blood and FDS tendons from horses, the study compared three PRP types: leukocyte-reduced, intermediate, and leukocyte-concentrated, with Dulbecco’s Modified Eagle Medium (DMEM) as a control. Excised tendons were cultured in these media, and after 72 hours, gene expression of COL1A1, COL3A1, COMP, matrix metalloproteinase (MMP)-13, IL-1β, and TNF-α was measured. The results showed no differences in COL1A1, COL3A1, or COMP expression among the PRP groups, although all exceeded control levels. MMP-13 expression was reduced in all PRP groups relative to the control. The inflammatory marker IL-1β was lowest in leukocyte-reduced PRP and highest in leukocyte-concentrated PRP, while TNF-α expression was minimal in leukocyte-reduced and control groups, and higher in intermediate and leukocyte-concentrated PRP. Overall, these findings suggest that higher WBC concentrations in PRP amplify the inflammatory response, which may promote scar tissue formation and potentially limit postoperative ROM due to adhesions [16].

Stem Cells

Shen et al. [30] investigated the effect of adipose-derived mesenchymal stromal cells (ASCs) on the inflammatory phase of intrasynovial tendon healing using 12 adult female mongrel dogs (Table 3).

**Table 3:** Characteristics of the in vivo animal studies using MSCs The table summarizes the outcome measures and key findings from animal studies evaluating the efficacy of MSCs in preventing postoperative adhesions following zone II flexor tendon repair. Results were considered statistically significant if the p-value <0.05. MRC1: Mannose Receptor C-Type 1; COL3A1: Collagen Type III Alpha 1 Chain; MMP3: Matrix Metalloproteinase-3; MSC: mesenchymal stromal cell.

Study	Species/ Sample Size	Intervention	Outcomes Measured	Key Findings
Shen et al. (2016) [30]	Adult female mongrel dogs, n=12	Adipose-derived MSC sheet applied around repair site	M2 macrophage stimulator genes (IL-4, IL-13, IL-10), M2 marker genes (MRC1, CD163), Scar associated genes (COL3A1, MMP3), Apoptosis genes (cleaved caspase-3)	Statistically significant increase in IL-4, IL-13, MRC1, CD163. Decreased COL3A1, MMP3, cleaved caspase-3.

All dogs included in the study underwent zone II transection of the second and fifth FDP tendons of the right forelimb, in the region between the annular pulleys, and were later repaired with a 4-0 Supramid core suture (S. Jackson, Inc., USA) and 6-0 Prolene (Ethicon, Raritan, New Jersey, USA) running epitenon suture. Following repair, all participants underwent two saline washes, and then five dogs received ASC sheets on both repaired digits (+ASC sheet group), while the remaining seven received HA alone on one digit (+HA group) and no additional treatment on the other digit, serving as HA and repair-only controls. All dogs then underwent the same postoperative and rehabilitation treatments. Seven days postoperatively, the dogs were euthanized, and tendon samples from the normal, repair-only, +HA, and +ASC sheet groups were analyzed for M2 macrophage stimulator genes (IL-4, IL-13, IL-10) and M2 marker genes (mannose receptor C-Type 1 (MRC1), CD163) to assess the inflammatory response, as excessive inflammation has been linked to increased fibroblast proliferation and adhesion formation. ASC treatment significantly upregulated IL-4 and IL-13 expression by 258-fold and four-fold, respectively, compared with the +HA and repair-only groups, which showed no significant changes. IL-10 expression remained unchanged across all groups. M2 marker genes MRC1 and CD163 were also elevated in ASC-treated tendons relative to other repair groups. Regarding tendon matrix remodeling, which is associated with scar formation and adhesions [30], ASC sheet treatment reduced expression of the scar-associated gene COL3A1 and decreased MMP3 expression compared with repair-only tendons. The study additionally examined cell apoptosis, believed to be related to scarring and adhesions [31], via cleaved caspase-3 immunostaining. ASC treatment reduced cleaved caspase-3 signals in both analyzed regions, suggesting decreased apoptosis. The reported findings of this study suggest that ASC sheets may modulate inflammation, reduce scar-associated matrix remodeling, and limit apoptosis, potentially mitigating adhesion formation during tendon healing [30].

Hyaluronic Acid

Isık et al. [32] investigated the efficacy of HA in reducing postoperative adhesions and improving ROM using thirty Leghorn chickens weighing 1000-1500 g (Table 4).

**Table 4:** Characteristics of the in vivo animal studies using HA The table summarizes the outcome measures and key findings from animal studies evaluating the efficacy of HA in preventing postoperative adhesions following zone II flexor tendon repair. Results were considered statistically significant if the p-value <0.05. HA: hyaluronic acid; CMC: carboxymethylcellulose; cd-HA: Carbodiimide-derivatized hyaluronic acid.

Study	Species/ Sample Size	Intervention	Outcomes Measured	Key Findings
Isık et al. (1999) [32]	Leghorn Chickens, n=30	HA-CMC membrane wrapped around the repair site, 0.2 ml NaHe injections	Adhesions, Joint Movement Peaks, Toe Displacement Curves	Unable to perform statistics on adhesions, statistically significant improvement in joint movement and toe displacement
Salti et al. (1992) [33]	New Zealand White rabbits, n=13	Low (0.1mg HA/1mL), medium (0.5mg HA/1mL), high (1.0mg HA/1mL) in culture	Tendon modulus, Apparent stress/strain, Energy absorption	No statistically significant difference in tendon modulus, apparent stress/strain, and energy absorption.
Zhao et al. (2010) [34]	Mixed-breed adult Dogs, n=60	95% solution composed of 1% sodium hyaluronate, 10% gelatin, and 1% carbodiimide hydrochloride + 0.2 mL of lubricin applied to tendons intraoperatively	Adhesion incidence, Work of flexion	Statistically significant reduction in adhesion formation and decrease in work of flexion
Zhao et al. (2013) [35]	Purpose-bred dogs, n=14	cd-HA–Lubricin coating	Adhesion grading, Work of flexion	Statistically significant decrease in adhesion scores

Each bird underwent a 75% partial laceration of the flexor tendon in zone II of digits two through four, followed by tendon repair using a modified Kessler technique with 5-0 nylon. For treatment, an HA-membrane, a slow-release HA formulation combined with carboxymethylcellulose (CMC), was wrapped around the repair site of the third toe. The second toe received 0.2 ml of NaHe (gel form of sodium hyaluronate), and the fourth toe received an equal volume of physiologic saline. All animals underwent identical postoperative care. Ten chickens were sacrificed each month for three months and evaluated histologically for adhesions as well as biomechanically through differential joint-movement peaks and toe-displacement curves. Histologic analysis showed dense fibrotic adhesions between the tendon and peritendinous tissues in the saline-treated groups at all time points. The HA-membrane and HA-treated tendons exhibited fibrotic bundles; however, they were arranged parallel to the direction of tendon gliding and the adhesions in the HA-membrane group appeared thinner and less cellular. Unfortunately, the authors were unable to perform statistical comparisons. Biomechanically, treatment groups were compared to the non-operated toes. Dynamic angular displacement patterns in the non-operated toes showed that applied force first mobilized the DIP joint, producing larger initial angular changes in the DIP than the PIP joint. This normal pattern was closely reproduced in only the HA-membrane group by the third postoperative month. In contrast, the NaHe and saline groups showed early motion of the PIP joint instead of the DIP, indicating that restrictive adhesions diverted force into surrounding tissues, limiting proper tendon glide. These groups also exhibited minimal differential peak values and shortened joint-motion duration, reflecting severely restricted excursion. Dynamic tip-displacement curves further supported these findings as by three months, HA-membrane and HA-treated toes closely resembled non-operated controls, whereas saline-treated toes did not. Quantitative analysis demonstrated significantly better motion in the HA-membrane and HA groups compared with saline (P=0.020). Moreover, both HA-based treatments showed increasing tip motion over time, indicating progressive maturation and improving functional recovery at the repair site (P=0.037) [32].

An additional randomized prospective control trial was performed to investigate the role of HA in preventing postoperative adhesions and diminished ROM. The study used 78 flexor tendons from 13 New Zealand white rabbits, weighing 2.5-3.5 kg, who underwent transection of their flexor tendons at zone II midpoints (Table 4). Tendons were divided into right and left forepaw groups and cultured for eight weeks in a medium consisting of 0.2 g HA to 150 mL of minimum essential medium. To minimize experimental bias, researchers divided each left and right forepaw group into a control group, low-concentration HA group (0.1 mg HA/1 mL solution), medium concentration HA group (0.5 mg HA/1 mL solution), and high concentration HA group (1.0 mg HA/1 mL solution). All rabbits included in the study underwent the same surgical repair, single simple volar and a single simple dorsal 9-0 nylon suture repair, and postoperative treatment. Although the study did not directly assess postoperative adhesions or ROM, it did evaluate tendon modulus, a measure of tendon stiffness that may indirectly influence adhesion formation by reducing tendon excursion. Tendon modulus increased with higher HA concentrations, but no statistically significant differences were found between the HA-treated groups and the control group. Additionally, while not the main focus of this review, the study also did not find a statistically significant difference among the groups in regards to apparent stress, apparent strain, and energy absorption [33]. 

Although Zhao et al. did not examine isolated HA in preventing postoperative zone II flexor tendon adhesions, they conducted studies evaluating carbodiimide-derivatized hyaluronic acid (cd-HA) and gelatin with added lubricin (CHL), as well as cd-HA combined with lubricin (cd-HA-Lubricin). In their 2010 randomized prospective controlled trial, 60 mixed-breed adult dogs weighing 20-25 kg underwent complete laceration of the second and fifth FDP tendon, 5 mm distal to the proximal digital flexor pulley within zone II, through a lateral longitudinal incision (Table 4). All tendons were then repaired with use of a two-strand modified Pennington technique with number-3-0 Ethibond sutures and reinforced with a simple running circumferential epitenon suture of 6-0 nylon. Following repair, the tendons were coated with a 95% solution composed of 1% sodium hyaluronate, 10% gelatin, and 1% carbodiimide hydrochloride (EDC). In the treatment group, an additional 0.2 mL of lubricin (260 μg/mL) was applied to the tendon surface, whereas tendons in the control group were rinsed only with saline. All animals followed the same postoperative and rehabilitation regimens. Outcome measures included in the study were gross evaluation of adhesions, work of flexion to assess digital function, gliding resistance to evaluate tendon surface properties, and mechanical strength and stiffness. The authors reported that the work of flexion in CHL-treated repaired tendons was significantly lower than in untreated repaired tendons at all time points (p<0.05). Additionally, adhesions were observed in 16 of 48 tendons (33%) in the CHL group compared with 31 of 55 tendons (56%) in the control group (p<0.05), demonstrating a statistically significant reduction in adhesions in the CHL-treated tendons by day 42 [34].

In their later study, Zhao et al. used 14 purpose-bred dogs (average weight 20 kg) that underwent laceration of the FDP tendons in zone II of the second and fifth digits (Table 4). The tendons were transected at the PIP joint level and repaired using a modified Kessler technique. Researchers then assessed the repaired tendons using a work-of-flexion score and an adhesion scoring system. Adhesions were graded at two locations: between the tendon and the pulley/sheath, and between the tendon and the tendon bed. The adhesion scale was defined as follows: zero - no adhesions, one - light adhesions (<5 mm in length, easily ruptured), two - moderate adhesions (5-20 mm, separable), three - severe adhesions (>20 mm, separable), and four - very severe adhesions (>20 mm, not separable). The study found that mean adhesion scores in zone II were significantly lower in the cd-HA-Lubricin-treated tendons compared with the saline control group (p<0.001) [35].

Investigation into the effects of HA on preventing postoperative adhesions in humans was performed in a randomized, double-blind, placebo-controlled trial [32]. The study included 22 patients with zone II flexor tendon lacerations involving both FDP and FDS tendons and monitored the effect of HA injections on peritendinous adhesions (Table 5).

**Table 5:** Characteristics of the in vivo human studies using HA The table summarizes the outcome measures and key findings from human studies evaluating the efficacy of HA in preventing postoperative adhesions following zone II flexor tendon repair. Results were considered statistically significant if the p-value <0.05. HA: hyaluronic acid; TAM: total active motion; TPM: total passive motion; DIP: distal interphalangeal joint; PIP: proximal interphalangeal joint; DIPAM: Distal interphalangeal joint active motion; QuickDASH: Quick Disabilities of the Arm, Shoulder, and Hand.

Study / Year	Sample size	Intervention	Outcomes measured	Key findings
Özgenel et al. (2012) [36]	n=22 patients	3 HA injections around tenorrhaphy site	TPM, TAM	No statistically significant difference in TPM or TAM at three weeks. Statistically significant increase in TPM and TAM at three months and long term.
Hagberg (1992) [37]	n=99 digits	0.4ml HA injection via catheter into the closed tendon sheath	TAM, PIP/DIP extension deficits, DIPAM	No statistically significant difference among TAM, PIP/DIP extension deficits, or DIPAM.
Riccio et al. (2009) [38]	n=45 patients	Hyaloglide gel applied intraoperatively	TAM, Functional grade, QuickDASH	Statistically significant increase in TAM in Hyaloglide group at 30, 60, 90, 180 days. Statistically significant increase in functional outcomes at 60, 90, 180 days. No statistically significant difference in QuickDASH scores.

Participants were randomly divided into two groups: 11 treated with three injections of HA around the tenorrhaphy site and 11 treated with saline in the same way. All participants in the study underwent a modified Kessler technique with a running epitendinous suture for repair of FDP and FDS tendons. All participants also underwent the Kleinert rehabilitation protocol. The researchers then investigated total active motion (TAM) and total passive motion (TPM) ROM at three weeks, three months, and long-term follow-up as an indirect measure of adhesion formation. In the saline-treated group, TPM measured 155.9° at three weeks, increasing to 177.7° at three months and 181.8° at long-term follow-up. In contrast, the HA-treated group demonstrated TPM values of 167.7° at three weeks, increasing to 212.3° at three months and 217.7° long-term. TAM values followed a similar pattern: the saline group improved from 107.3° at three weeks to 141.4° at three months and 147.7° at long-term, whereas the HA group improved from 118.6° at three weeks to 166.4° at three months and 176.4° long-term. Although no statistically significant differences in TPM or TAM were observed at the three-week evaluation (p>0.05), both measures were significantly higher in the HA-treated group at three months and long-term (p<0.05), demonstrating improved functional outcomes and suggesting reduced adhesion formation with HA use [36].

Further investigation into the efficacy of HA in preventing adhesion formation following zone II flexor tendon repair by conducting a prospective, double-blind, randomized clinical study using 120 human digits (Table 5). Prior to the start of the study, the digits were stratified into six different classes (1) to separate two different types of surgical treatment (tenorrhaphy and two-stage tendon grafting), (2) to separate digits with complete FDS severance from those with only partial or no FDS injury, and (3) to classify the digits subjected to tendon grafting according to whether there were additional injuries that may influence the functional recovery. Within each class, the digits were randomly allocated into two different treatment groups, one was the experimental group which received 0.4 ml injection of HA in the form of sodium hyaluronate via catheter insertion into the closed tendon sheath and the control group which was treated with an equivalent volume of physiologic saline solution and administered the same way as above. All surgically treated digits had similar postoperative care and all hands were immobilized in a dorsal splint with the wrist and MCP joints in flexion. On day three following surgery, controlled mobilization of the injured finger was started according to the method of Kleinert and continued for five weeks. All digits that maintained inclusion criteria during the follow up period (99 digits) were evaluated on three major criteria at least four months after surgery: TAM of the treated digit (MCP + PIP + DIP included); extension deficits (ext. def.) of PIP and DIP joints; and the DIPAM. Researchers found no statistically significant differences between treatments in any of the studied variables for any class of tendon injury (TAM: p=0.74; ext. def.: p=0.82; DIPAM: p=1) [37]. 

Lastly, a multicenter randomized controlled trial was conducted to evaluate whether Hyaloglide, a cross-linked HA gel, could reduce postoperative adhesions in patients undergoing zone II flexor tenolysis after failed flexor tendon repair (Table 5). The study included 45 participants, 19 of which were assigned to the control group receiving standard repair and 26 were assigned to the Hyaloglide group. All participants in the study underwent the same surgical technique by surgeons of similar experience and same postoperative care. Rehabilitation protocols varied based on whether the A2 and A4 pulleys required repair; however, were the same among those in each group. Application of the Hyaloglide gel was applied after the tourniquet was released and hemostasis was achieved, just prior to skin closure [38]. The gel was applied along the exposed tendon surface and in the digital tube to cover the entire surgical area. Outcomes were assessed at 30, 60, 90, and 180 days postoperatively and those relevant to this review included TAM, functional grade, and the Quick Disabilities of the Arm, Shoulder and Hand (QuickDASH) questionnaire which accounted for subjective reported disability outcome following surgery. Adhesions were identified in the right hand of 12 patients in each group. In most cases, the second finger was involved. Adhesions affected both the deep and superficial flexor tendons in 14 patients (74%) in the control group and 15 patients (58%) in the Hyaloglide group, while the remaining five (26%) and 11 (42%) patients, respectively, exhibited adhesions of the deep flexor tendons only. Although preoperative TAM was similar between groups, by postoperative day 30, patients treated with Hyaloglide demonstrated significantly greater TAM than controls (56 vs. 39; P=0.01). The largest group differences, which were statistically significant, occurred at T30 (10% vs. 27%; P=0.006) and T90 (20% vs. 41%; P=0.007). Functional outcomes followed a similar pattern: At baseline, all fingers in both groups demonstrated fair or poor function. By T30, 31% of Hyaloglide-treated fingers achieved good or excellent function, compared with 5% in the control group (P=0.06). This difference reached statistical significance at T60 (50% vs. 11%; P=0.009) and remained significant at T90 and T180 in favor of Hyaloglide. In contrast, QuickDASH scores did not differ significantly between groups at any time point [38].

Discussion

To our knowledge, this is the first review to comprehensively synthesize the available literature on the use of PRP, MSCs, and HA in preventing postoperative adhesions following zone II flexor tendon repair [29-38]. The current evidence is mixed: some studies indicate that these biologic adjuncts may reduce adhesion formation, while others report no statistically significant benefit over standard surgical and rehabilitation protocols. This review also examines some of the proposed mechanisms underlying their effects; however, it highlights the lack of a well-established biological explanation for how these agents might prevent postoperative adhesions in zone II of the volar hand. 

We further examine the complex anatomy of zone II, historically termed “no man’s land,” and the challenges it presents for flexor tendon repair. By detailing the high incidence of complications, including adhesion formation and reduced ROM, we highlight the continued need for research into strategies that may enhance healing and improve functional outcomes. Given the wide range of conflicting findings across both human and animal studies, this review supports ongoing exploration of these agents, as well as alternative biologic or mechanical approaches, for preventing postoperative adhesions in zone II repairs. Overall, the collective findings suggest that none of the investigated agents can consistently or reliably prevent postoperative adhesions in zone II of the hand at this time, despite their documented roles in treating other tendon pathologies.

Several factors may contribute to the variable outcomes reported in the literature, including differences in patient age, surgical techniques, rehabilitation protocols, and patient adherence to postoperative therapy. Perhaps most importantly, the inherent complexity of zone II anatomy may limit the consistency and effectiveness of any intervention aimed at reducing adhesions. Continued, well-designed research is essential to determine whether any treatment can reliably prevent postoperative adhesion formation in this anatomically challenging region.

Although the current literature does not support the use of PRP, MSCs, or HA for consistently preventing postoperative adhesions in zone II flexor tendon repairs, some studies report promising outcomes that warrant further investigation. However, several limitations must be acknowledged. This review focuses specifically on zone II of the volar hand, a region known for its complex anatomy and high complication rates. Thus, while the findings do not support routine use of these agents in zone II, they do not preclude potential benefits in other flexor tendon zones or other anatomical regions. Additionally, some included studies are dated, and surgical techniques, research methods, and rehabilitation protocols may have evolved considerably over time, emphasizing the need for updated comparative studies. Finally, the wide age ranges represented within the study populations may have influenced outcomes, as studies reporting positive effects may have included younger patients who were more responsive to these treatments.

Another important consideration when evaluating the clinical applicability of these agents, or any agents moving forward, is their cost. Questions remain regarding whether these treatments are financially feasible for patients across diverse backgrounds and demographics, whether they are covered by insurance plans, and how accessible they are to individuals without insurance. These factors must be carefully examined before any of these adjuncts can be recommended for widespread use in preventing postoperative adhesions in zone II of the hand.

## Conclusions

This review evaluated the postoperative benefits and clinical efficacy of PRP, MSCs, and HA in reducing adhesion formation and improving ROM following Zone II flexor tendon repair. Based on the available randomized controlled trials and prospective and retrospective cohort studies in both human and animal models, current evidence does not demonstrate consistent or reliable improvements in postoperative adhesion prevention or functional ROM with these biologic adjuncts.

These findings highlight the need for further studies to clarify their therapeutic role in the setting of zone II flexor tendon repair. Future research should focus on age-specific patient populations, optimization of biologic formulation and delivery methods, and improved mechanistic understanding of adhesion modulation. Given the anatomic complexity and high complication rates associated with zone II repairs, continued investigation into both biologic and mechanical strategies for adhesion reduction remains warranted.
